# HFD aggravated the arthritis and atherosclerosis by altering the intestinal status and gut microbiota

**DOI:** 10.1186/s10020-024-01014-3

**Published:** 2024-12-23

**Authors:** Na Shi, Shan Jiang, Yue Zhao, Yang Zhang, Xinwang Duan, Guo-bao Hong, Zhongshan Yang, Yuanyuan Duan, Haitao Niu

**Affiliations:** 1 Key Laboratory of Viral Pathogenesis and Infection Prevention and Control (Jinan University), Ministry of Education, School of Medicine, Jinan University, Guangzhou, 510632 China; 2 Guangzhou Key Laboratory for Germ-Free Animals and Microbiota Application, School of Medicine, Institute of Laboratory Animal Sciences, Jinan University, Guangzhou, 510632 China; 3 Yunnan Provincial Key Laboratory of Molecular Biology for Sinomedicine, School of Basic Medical Sciences of Yunnan University of Chinese Medicine, Kunming, Yunnan China; 4 Department of Cardiology, Peking Union Medical College Hospital, Beijing, China; 5 Department of Rheumatology and Immunology, The Second Affiliated Hospital, Jiangxi Medical College, Nanchang University, Nanchang, 330006 Jiangxi China; 6 Department of Nephrology, The Affiliated Shunde Hospital of Jinan University, Guangdong, China

**Keywords:** Arthritis combined with atherosclerosis, Inflammation, Gut microbiota, Intestinal status

## Abstract

Rheumatoid arthritis (RA) and cardiovascular disease (CVD) are both the chronic inflammatory disease. To investigate the influence of secondary atherosclerosis on arthritis mice, we treated the ApoE^−/−^ mice with K/BxN serum and high fat diet (HFD), and subsequently assessed the phenotypes as well as immune profiles of K/BxN serum and HFD induced ApoE^−/−^ mice. We found that HFD treatment aggravated the hyperlipidemia, atherosclerotic lesions, ankle swelling and arthropathy of mice. We further demonstrated that HFD altered the gut microbiota and metabolism, intestinal homeostasis and Th17/Treg cell balance in lamina propria lymphocytes. Moreover, HFD decreased the number of Peyer’ s patches and altered the expression profiling of gut immune cells. In addition, HFD increased the number of aortic leukocytes and macrophages, then aggravated the atherosclerosis in aorta, which led to greater inflammation in mice aorta and aortic root. Collectively, our study indicated that HFD aggravated the arthritis and atherosclerosis, which may be contributed by microbiota dysbiosis, the intestinal permeability and disrupted immunological homeostasis.

## Introduction

Rheumatoid arthritis (RA) is a chronic autoimmune disease characterized by synovial inflammation and autoimmune antibody (Kishikawa et al. 2020). In recent years, significant progress has been made in the treatment of joint symptoms in RA patients, but the mortality rate remains higher than the general population. Atherosclerosis is the main cause of cardiovascular disease (CVD) and is a chronic inflammatory disease related with innate and adaptive immune system both involving in the progression of plaques (Dong et al. 2022). RA and atherosclerosis share the common pathophysiologic mechanisms and pro-inflammatory cytokines could promote both of them simultaneously (Kerola et al. 2021; Karpouzas et al. 2021). Rheumatoid synovitis and unstable atherosclerotic plaques secrete the pro-inflammatory cytokines, including interleukin (IL)-6, IL-1 and tumour necrosis factor (TNF)-α (Kerola et al. 2021). IL-1α is associated with infarct size, whereas IL-1β is related to adverse cardiac remodeling (Kerola et al. 2021). TNF-α regulates the vascular homeostasis and predicts the risk of future cardiovascular events. IL-6 not only involves in local tissue and systemic inflammation, but also promotes the differentiation of naïve T-helper cells into Th17 cells (Stephenkaptoge et al. 2014). Previous studies have suggested that atherosclerosis is caused by successive lipid deposition in artery walls. Recently it is believed to result from the gut microbiota disorders (Dong et al. 2022).

The gut microbiota is comprised of trillions of non-pathogenic microbes, which play a crucial role in the digestion and absorption of nutrients, remarkably modulate the immune system and metabolism, and have influence on human health (Kishikawa et al. 2020; Wang et al. 2011). Over 90% microbiotas in gastrointestinal tract belong to *Firmicutes* and *Bacteroidetes* phyla (Dhaliwal 2019). The subdominant phyla includes *Proteobacteria*, *Actinobacteria*, and *Verrucomicrobia *(Magne et al. 2020). The previous studies have shown that different gut microbiota communities play vital roles in various inflammatory diseases, such as RA, atherosclerosis, CVD, type 2 diabetes, and inflammatory bowel diseases (Kishikawa et al. 2020; Wang et al. 2011; Maeda et al. 2016; Xu et al. 2022). The gut microbiota metabolites and bacteria are closely associated with the pathogenesis of CVD, particularly under conditions of dysbiosis (Xu et al. 2022; Nesci et al. 2023; Huang et al. 2022). The gut microbiota is correlated with inflammation on account of the lipopolysaccharide (LPS) and platelet hyperactivation mediated by TMAO. As known to all, LPS is a Gram-negative bacterium and a trigger of systemic inflammation. It contributes to the development of atherosclerosis due to its association with the formation of foam cells and the accumulation of cholesteryl ester. Meanwhile, LPS promotes the secretion of pro-inflammatory cytokines and the TMAO precursor, trimethyllysine (TML) (Nesci et al. 2023).

The animal studies have revealed that *Prevotella copri* precipitates susceptible mice developing arthritis (Maeda et al. 2016). The gut microbiota of patients with arterial hypertension shows an increased abundance of *Clostridiales* and *Bacterodiales* both in male human and mice models (Nesci et al. 2023). *Subdoligranulum didolesgii* triggers joint synovitis of germ-free DBA/1 mice and the deposition of complement and immunoglobulins (Kriegel 2023). The gut microbiota could trigger a systemic inflammatory response including CVD. Thus, specific therapeutic modulation may potentially reduce CVD risk, such as dietary intervention, probiotics, drugs, small molecule antimicrobial enzyme therapeutics and faecal microbiota transplantation (Nesci et al. 2023).

Arthritis combined with atherosclerosis seriously impairs the life quality of sufferers. Our study characterized the influence of HFD on arthritis mice and the relationship between gut microbiome and arthritis combined with atherosclerosis through a murine model. Our results provided insights for further study and lay the experimental and theoretical basis of this disease.

## Materials and methods

### Ethics approval and consent to participate

Human faeces were obtained in accordance with the local ethics committees (JS-1195, Peking Union Medical College Hospital, Beijing), and informed consent were obtained from all subjects. The faeces were obtained from 27 healthy volunteers, 22 arthritis patients combined with atherosclerosis, 15 arthritis patients and 14 atherosclerosis patients. Arthritis patients conformed with the diagnostic criteria of rheumatoid arthritis. Arthritis patients combined with atherosclerosis conformed with the diagnostic criteria of atherosclerosis associated with rheumatoid arthritis. Atherosclerosis patients exhibited ≥ 50% stenosis in at least one main coronary artery. And in the past months, they underwent initial treatment or low to medium dose hormone maintenance therapy. The subjects in controls were those who exhibited negative results upon coronary angiography examination or had no CAD and rheumatoid arthritis related clinical signs and symptoms. Subjects were excluded if they had malignant tumours, infectious diseases, gastrointestinal diseases, pregnancy or lactation, took antibiotics for more than 3 days in the previous 3 months or had a history of gastrointestinal surgery in the previous years.

### 16S rDNA sequencing for human faeces bacterial profiles and analysis

The faeces of arthritis patients combined with atherosclerosis, arthritis patients, atherosclerosis patients and healthy human were collected and total DNA were extracted by CTAB/SDS method. Then the V4 region was amplified by PCR, prepared for library built using TruSeq® DNA PCR-Free Sample Preparation Kit, quantified by Qubit and Q-PCR, and then sequenced by Illumina HiSeq2500 PE250. Sequences were analyzed by Uparse software (Uparse v7.0.1001) and those with 97% identity threshold were clustered into Operational Taxonomic Units (OTUs) using UPARSE pipeline. The representative sequences were assigned to a taxonomic identity using RDP classifier based on a default confidence threshold of 0.8–1.0. The observed-species, Chao1, Shannon, Simpson, ACE, Goods-coverage and PD_whole_tree index were calculated using Qiime pipeline. Then the Alpha diversity index and Beta diversity index differences between groups were analyzed by QIIME (Version1.7.0) and displayed with R software. t-test and Wilcox were utilized to test differences between two groups, and Tukey and Wilcox were employed to test among three groups and more. Lefse analysis and Metastats were carried out respectively by LefSe software and R software. All sequencing and analysis were performed by Novogene, Beijing, China.

### Animal model

Apolipoprotein E deficient (ApoE^−/−^) mice with a C57BL/6 background were purchased from Beijing Vital River Laboratory Animal Technology Co., Ltd. KRN mice were provided as gifts from Dr. Diane Mathis and Christoph Benoist’ s colony at Jackson Laboratory, USA. NOD/LtJ mice were purchased from HuaFuKang Bioscience Co., LTD, Beijing. The K/BxN mice were homozygous cross of the KRN mice and the NOD/LtJ mice carrying the Ag7 allele. The first-generation K/BxN mice expressed both the T cell receptor (TCR) transgene KRN and the MHC class II molecule Ag7 developing severe inflammatory arthritis. Serum from these mice causes similar arthritis in a wide range of mouse strains, due to the fact that autoantibodies recognize glucose-6-phosphate isomerase (GPI). Mice were housed in IVC laboratory and fed on chow diet until 8 weeks old. Institute of laboratory animal sciences, Chinese academy of medical sciences Animal Care and Use Committee approved all animal procedures [ILAS-PG-2015-002].

### Induction the symptom of arthritis combined with atherosclerosis

Mice were weaned at 4 weeks old. At 8 weeks, mice were divided into 2 groups and injected with 100μL K/BxN serum and C57BL/6 serum every 2 weeks respectively. Mice injected with K/BxN serum developed joint swelling within 1 week, while those injected with C57BL/6 serum did not. Instead of normal chow diet (CD), part of mice injected with K/BxN serum were treated with high fat diet (HFD) from Special Diets Services (Bo Tai Hong Da, Beijing) consisting of (w/w) fat (15.8%), cholesterol (1.25%) and cholate (0.5%) to induce pathological atherosclerosis after the joint swelling. Mice injected with C57BL/6 serum experienced the same operation procedures at the same time point. Mice were randomly divided into four different groups. Mice in the + K/BxN serum + HFD group (n = 9), while mice in the + K/BxN serum-HFD group (n = 11). Mice in the −K/BxN serum + HFD group (n = 15), while mice in the −K/BxN serum −HFD (n = 11). Arthritis severity was jointly determined by thickness change of the hind paw ankle joints and the clinical score (total = 12) for all fore paws, where 0 = normal, 1 = swollen wrist, 2 = swelling extending to dorsal paw, 3 = swelling extending to the digits. Ankle thickness was measured weekly utilizing a caliper placed across the ankle joint at the widest point of both hind limbs.

### Analysis of serum total cholesterol, oil red O staining of aortic root and HE staining of ankle joints

Mouse blood was obtained from the cheeks before euthanization. Serum was collected by centrifugation. Total cholesterol in serum was measured using enzymatic assays in accordance with the manufacturer’s instructions (abcam). Then mice were euthanized at 24 weeks old. Tissues were well perfused in situ with 15 mL saline and a cannula was inserted into the left ventricle to remove the blood cell. The aortic roots were embedded, sectioned and stained with oil red O under the help of optical coherence tomography. Starting from the 3 valve cusps of the aortic sinus aortc, root were cut into 7-μm sections with 35-μm intervals by freezing microtome (Leica), and stained with oil red O (sigma) for atherosclerotic plaque measurement. Images captured with camera (Leica), magnified 40 × , were utilized for achieving the percentage of oil red O stained lesion area (calculated by oil red O stained area/ the cross-sectional area of the aorta × 100) by Image-Pro Plus 6.0 software (Media Cybernetics Inc.). Ankle joints were harvested, fixed in 10% formalin and decalcified, paraffon embedded, sectioned and stained with HE.

### 16S rDNA sequencing for mice faeces bacterial profiles and analysis

Mice faeces were collected and total DNA was extracted by CTAB/SDS method. 16S rDNA was sequenced through the same methods as human faeces.

### RNA extraction and transcriptome sequencing for mice colon

Mice colons were collected and total RNA was isolated using Trizol reagent (Invitrogen) according to manual instruction. Qualitative and quantitative analysis of total RNA was conducted through the Agilent 2100 bioanalyzer (Agilent Technologies) and Nano Drop (Thermo). Total RNA of each sample was reverse-transcribed to cDNA and amplified by polymerase chain reaction (PCR). Sequencing libraries were generated by NEBNext®UltraTM RNA Library Prep Kit for Illumina® (New England Biolabs) and amplified with phi29. The library was sequenced in MGISEQ2000 sequencing platform and 125/150 bp paired-end reads were generated.

Raw data in the fastq format was filtered with SOAPnuke. After discarding the low quality reads, removing the reads containing adapter sequences and poly-N sequences from the raw data, high-quality clean reads were obtained and then aligned to gene set. Differential expression gene levels were determined using RSEM and all differential expression analysis was performed using the DESeq2 with Q value ≤ 0.05. Gene ontology (GO) and Kyoto Encyclopedia of Genes and Genomes (KEGG) enrichment analysis of annotated different expression gene was performed using Phyper based on Hypergeometric test. Terms and pathways of significance were corrected by Q value with a rigorous threshold (Q value ≤ 0.05) using Bonferroni.

### Metabolism analysis for mice faeces

Mice faeces from each group were collected. 0.1 g faeces was respectively surrounded with liquid nitrogen and the homogenate was well vortexed with prechilled 80% methanol and 0.1% formic acid for resuspension. The samples were incubated on ice for 5 min and then centrifuged at 15,000 rpm, 4 ℃ for 5 min. Part of supernatant was diluted with LC–MS grade water to obtain ideal concentration containing 60% methanol. The samples were subsequently transferred to a fresh Eppendorf tube through a 0.22 µm filter and then centrifuged at 15,000 rpm, 4 ℃ for 10 min. Finally, 100 µL liquid sample and 400 µL prechilled methanol were well mixed by vortex and then injected into the LC–MS/MS system for analysis.

LC–MS/MS analysis was performed using a Vanquish UHPLC system (Thermo Fisher) coupled with an Orbitrap Q Exactive series mass spectrometer (Thermo Fisher). The raw data files generated by LC–MS/MS were processed using the Compound Discoverer 3.1 to perform peak alignment, peak-picking, and quantitation for each metabolite. The normalized data were used to predict the molecular formula based on additive ions, molecular ion peaks and fragment ions. We applied univariate analysis (*t*-test) to calculate the statistical significance (*P*-value). The metabolites with VIP > 1 and *P*-value < 0.05 and fold change ≥ 2 or FC ≤ 0.5 were considered as differential metabolites. Volcano plots were used to filter metabolites of interested based on Log2 (FC) and − log10 (*P*-value).

### Immunofluorescence staining of aortic root

For immunofluorescence staining, 7 μm sections were blocked with 10% rabbit serum for 1 h and then incubated with antibodies. Plasma cells, M1 macrophages and macrophages were detected by biotin anti-mouse CD138, biotin anti-mouse CD11c, biotin anti-mouse CD11b respectively. HRP-Streptavidin and Alexa Fluor® 488 Tyramide TSA™ Kit (Invitrogen) were used to amplify the signal. B cells and IgG deposition were detected by Alexa Fluor® 594 anti-mouse CD19 and Alexa Fluor® 594 anti-mouse IgG respectively. All slides were mounted with prolong gold antifade reagent with DAPI (Invitrogen). All antibodies were purchased from Biolegend. Data were captured in images by camera (Leica), magnified 400 × and 1000 × , and then analyzed by Image-Pro Plus v6.0 software (Media Cybernetics Inc., USA). Images of representative aortic roots of every sample were captured under the same conditions of light intensity and exposure time. The deposition area was quantitatively analyzed by average integrated optical density parameter (mean IOD).

### Analysis of serum cytokines

Serum tumor necrosis factor-α (TNF-α), interleukin 6 (IL-6), IL-10, IL-1α, IL-2, IL-1β, IL-3, IL-4, IL-5, IL-9, IL-12 (p40), IL-12 (p70), IL-13, IL-17, Eotaxin, Granulocyte colony stimulating factor (G-CSF), Granulocyte–macrophage colony-stimulating factor (GM-CSF), Keratinocyte chemoattractant (KC), Monocyte chemoattractant protein-1 (MCP-1), Macrophage inflammatory protein-1α (MIP-1α), Macrophage inflammatory protein-1β (MIP-1β), RANTES and interferon-γ (IFN-γ) were analyzed via Luminex 200 Mouse Magnetic 23 plex Assay (Biorad) according to the manufacturer’ s instructions.

### Flow cytometry analysis of aortic cells and intestinal lymphocytes

To harvest aortic cells, the aortas from the aorta arch to the iliac artery were cut into 2 mm pieces and incubated in enzyme mixture (collegenase I, collegenase XI, hyaluronidase and Dase I in DPBS with Calcium and Magnesium) at 37℃ with gentle shaking for 90 min.

Peyer’s patch (PP) lymphocytes were minced. Lamina propria (LP) lymphocytes were prepared as previously described (Weigmann et al. 2007a; Weigmann et al. 2007b). Lamina propria lymphocytes were stained by intracellular antibodies and extracellular antibodies, while aortic cells were merely stained by extracellular antibodies. Cells were stained on ice with fluorochrome-conjugated monoclonal antibodies for 30 min. All antibodies were purchased from BD Biosciences (listed in Table 1). The percentage of cells was analyzed by flow cytometer (BD FACSymphony A5) and data were analyzed by flowjo v10.

**Table 1:** List of antibodies used in flow cytometry analysis

Antigen	Manufacturer	Conjugation
CD19	BD	BUV737
B220	BD	BUV395
CD138	BD	BV421
CD38	BD	PE
CD95	BD	BV510
IgM	BD	APC
IgD	BD	BV786
PNA	BD	FITC
CD103	BD	PE-CF594
CD11b	BD	APC-CY7
CD93	BD	BV650
CD5	BD	APC-R700
CD23	BD	PE-CY7
MHC2	BD	BV711
CD3e	BD	APC-CY7
CD4	BD	BUV395
CD8	BD	PercP-CY5.5
CD68	BD	PE
CD25	BD	BB515
CXCR3	BD	APC
RORγt	BD	BV786
Foxp3	BD	BV421
IL17A	BD	BV510
CD45	BD	BV605
CD11c	BD	APC
CD11b	BD	FITC

### Statistical analysis

The statistical analysis was done by GraphPad Prism software (San Diego, CA). The Mann–Whitney *t*-test was used for comparison. A *P*-value below 0.05 was considered significant.

## Results

### HFD aggravated the ankle swelling and arthropathy of arthritis mice combined with atherosclerosis

**Fig. 1:**
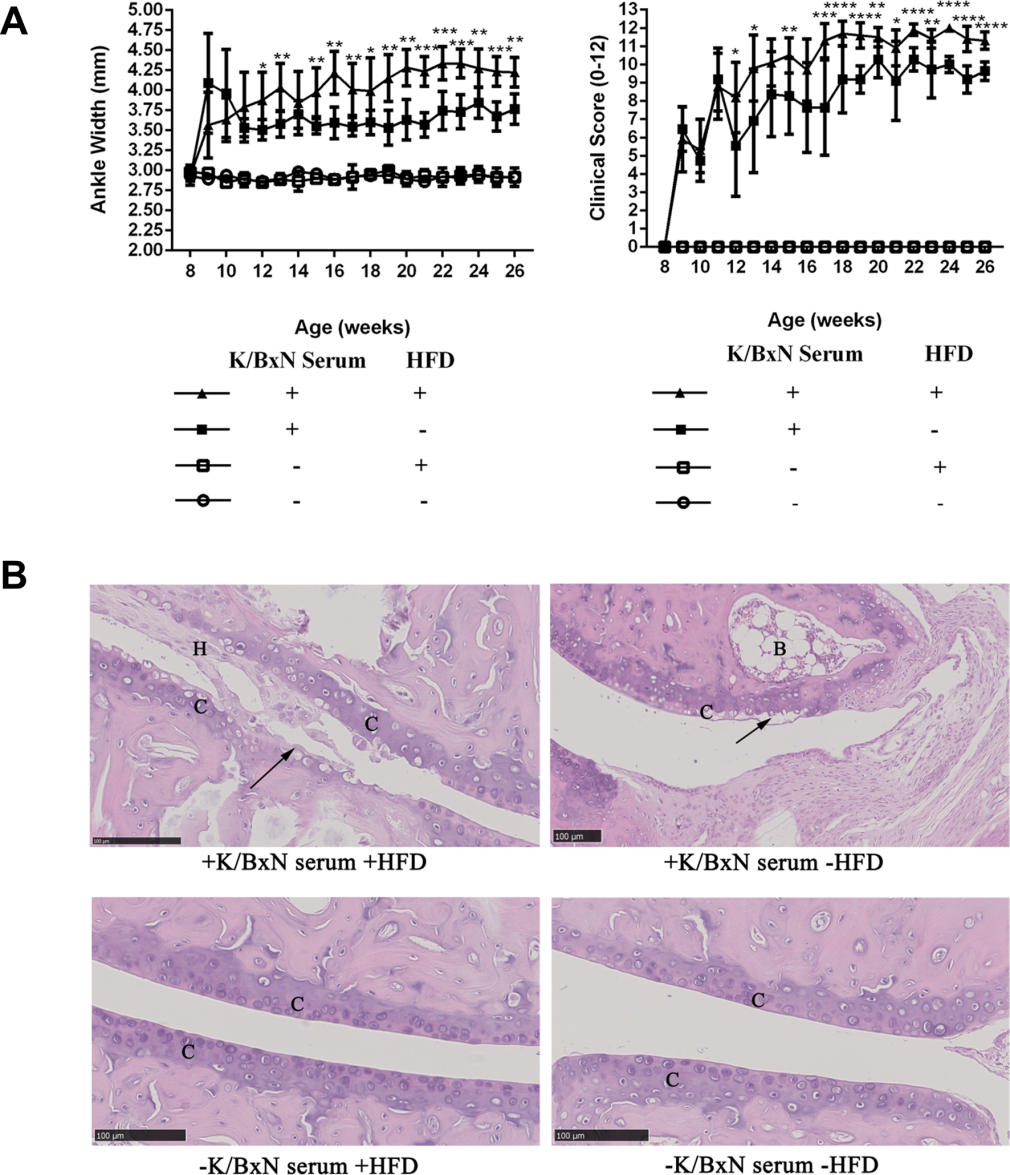
HFD aggravated the ankle swelling and arthropathy of arthritis mice combined with atherosclerosis. **A** The ankle width and clinical score of mice. **B** HE staining of representative ankle joint specimens in four groups. H = Hyperplasia, B = Bone marrow, C = Cartilage. Data were presented as mean ± SD. Asterisk denoted statistically significant differences: *P < 0.05, **P < 0.01, ***P < 0.001, ****P < 0.0001. Representative results of two independent experiments were shown

In order to define the phenotypes of arthritis mice combined with atherosclerosis, we applied ApoE^−/−^ mice with K/BxN serum injection and HFD feeding to this study. The ankle joint of mice swelled just 1 week after the K/BxN serum injection, then the swelling continued increasing after HFD feeding. The results of ankle width and clinical score indicated that compared with arthritis mice (+ K/BxN serum-HFD), the ankle swelling of arthritis mice combined with atherosclerosis (+ K/BxN serum + HFD) significantly augmented (Fig. 1A). It suggested that HFD may exacerbate the ankle bulk and the process which stimulated by K/BxN serum treatment.

HE staining showed that K/BxN serum and HFD treated mice developed severe and destructive arthropathy. HFD aggravated the chondrocyte apoptosis and brought some cells fall off, making the cartilage face of articular cavity no longer smooth as well as bulge or depression formed. Meanwhile the articular cavity produced a large number of hyperplasia (H) such as fibroblasts. In the articular cavity of mice which was simply injected with K/BxN serum, no hyperplasia was found, but lots of chondrocyte apoptosis were detected. Whereas the mice, which had not been injected with K/BxN serum regardless of HFD, presented smooth cartilage (C) surface without any damage in articular cavity, and orderly arranged chondrocytes (Fig. 1B).

### HFD worsened the atherosclerosis of arthritis mice combined with atherosclerosis

**Fig. 2:**
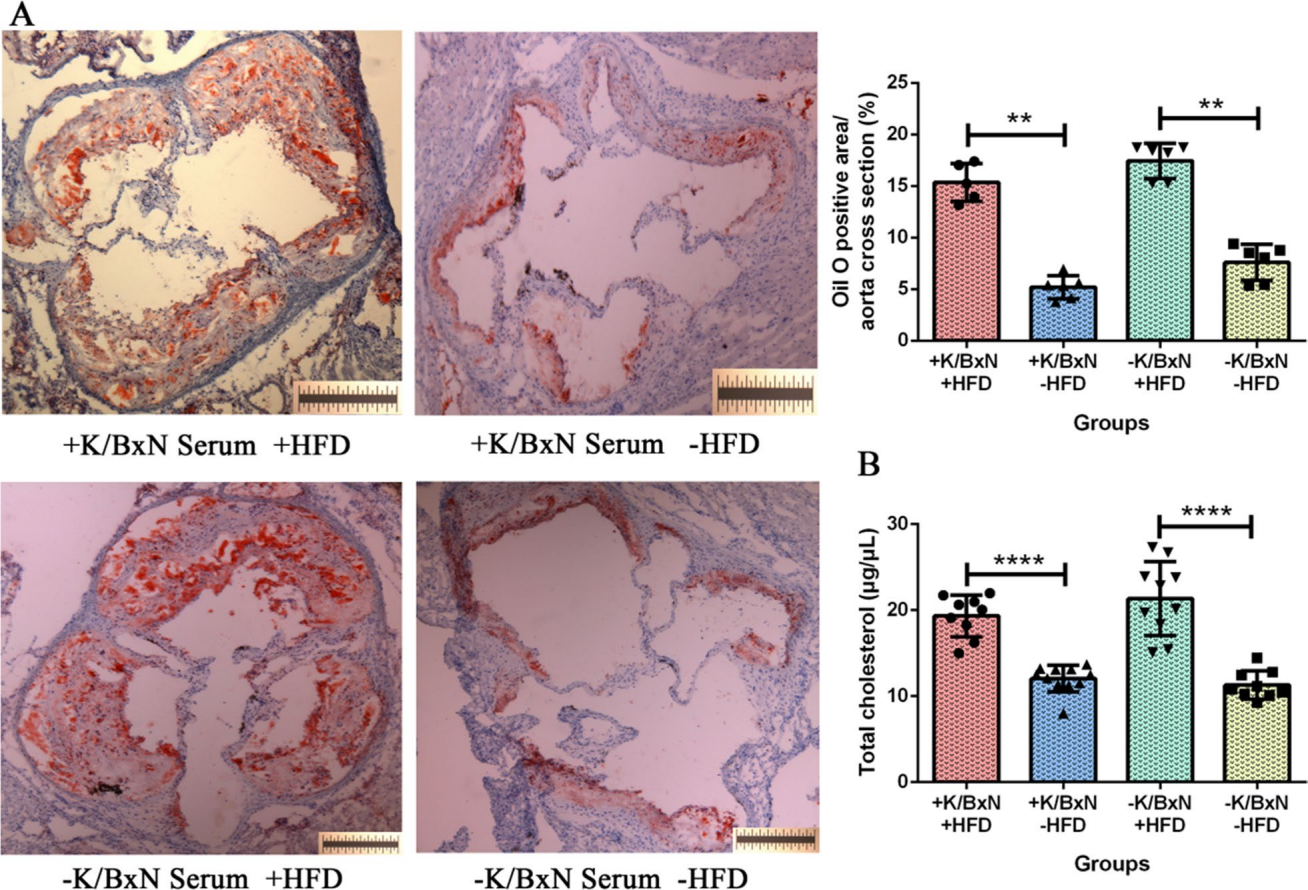
HFD worsened the atherosclerosis of arthritis mice combined with atherosclerosis. **A** The atherosclerotic plaques in cross-sectional area of the aortic root stained by oil red O and quantification of atherosclerosis per aortic root in four groups. **B** The content of total cholesterol (µg/µL) in serum of four groups. Data were presented as mean ± SD. Asterisk denoted statistically significant differences: *P < 0.05, **P < 0.01, ***P < 0.001, ****P < 0.0001. Representative results of two independent experiments were shown

To evaluate the effect of HFD on arthritis mice, atherosclerotic plaques in cross-sectional area of the aortic roots were analyzed by oil red O staining. The result of oil red O staining concluded that compared with arthritis mice (+ K/BxN serum -HFD), the atherosclerotic plaques in cross-sectional area of aortic roots were significantly augmented in arthritis mice combined with atherosclerosis (+ K/BxN serum + HFD) (*P* < 0.01). Compared with normal mice (−K/BxN serum -HFD), the atherosclerotic plaques in cross-sectional area of aortic roots were significantly augmented in atherosclerosis mice (−K/BxN serum + HFD) (*P* < 0.01) (Fig. 2A). It suggested that HFD expanded the area of atherosclerotic plaques.

To determine whether HFD treatment affected the lipid level, the concentration of serum total cholesterol was measured by enzyme-linked immunosorbent assay (ELISA). The result of serum total cholesterol indicated that compared with arthritis mice (+ K/BxN serum −HFD), arthritis mice combined with atherosclerosis (+ K/BxN serum + HFD) had a significantly higher level of total cholesterol (*P* < 0.0001). Compared with normal mice (−K/BxN serum −HFD), atherosclerosis mice (−K/BxN serum + HFD) had a significantly higher level of total cholesterol (*P* < 0.0001) (Fig. 2B). It suggested that HFD increased the level of serum total cholesterol of arthritis mice and non-arthritis mice. According to above analysis, arthritis mice combined with atherosclerosis were cultivated successfully.

### HFD generated greater inflammation of arthritis mice in aorta, aortic root and serum

**Fig. 3:**
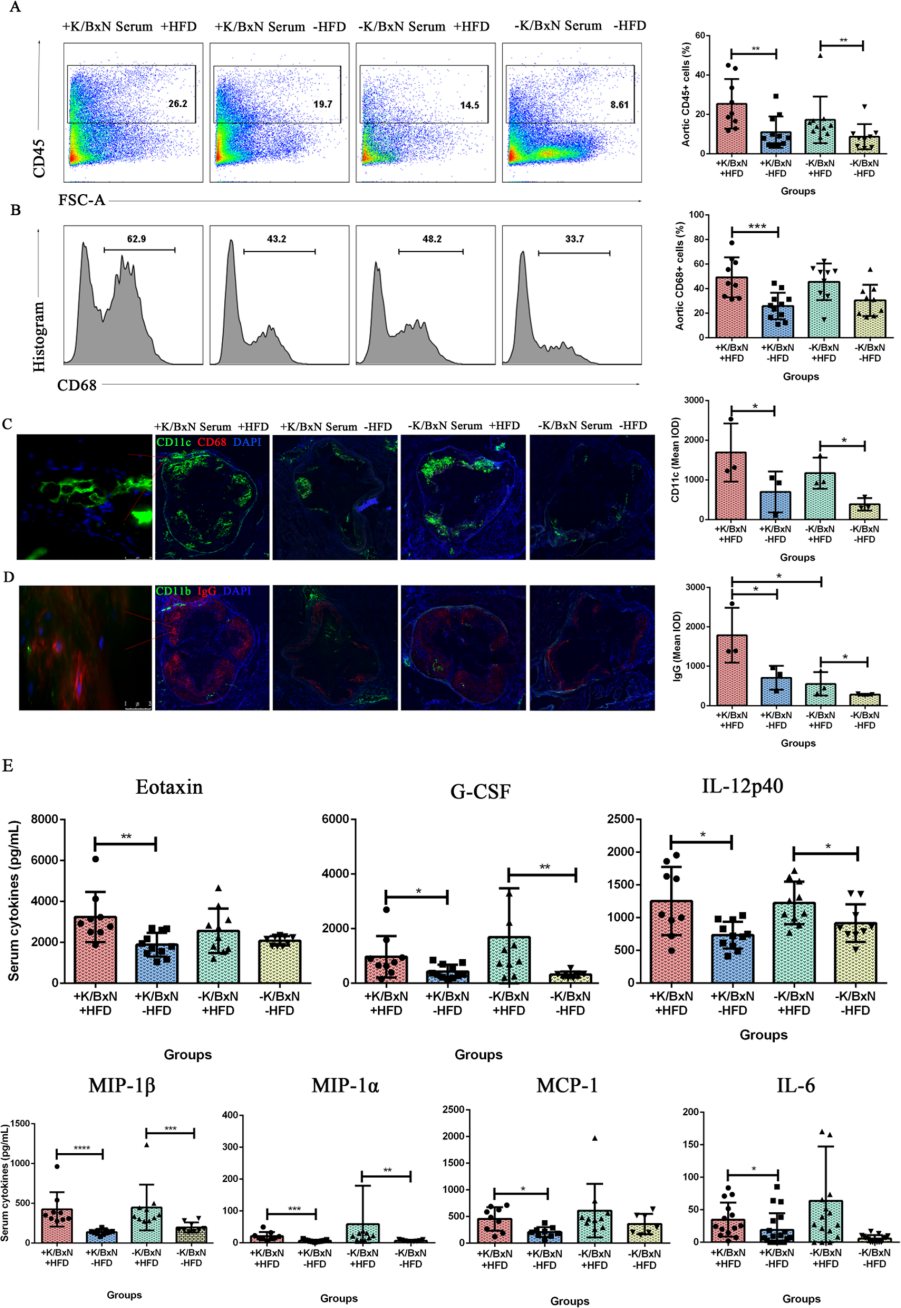
HFD generated greater inflammation in aorta, aortic root and serum of arthritis combined with atherosclerosis mice. **A** Flow cytometry gating strategy and the percent of vascular CD45^+^ leukocytes of aorta in four groups. **B** Flow cytometry gating strategy and the percent of vascular CD68^+^ macrophages of aorta in four groups. **C** Immunofluorescent staining and the mean IOD of CD11c. **D** Immunofluorescent staining and the mean IOD of IgG. **E** The content of serum cytokines (pg/mL). Data were represented as mean ± SD. Asterisk denoted statistically significant differences: *P < 0.05, **P < 0.01, ***P < 0.001. Representative results of two independent experiments were shown

The atherosclerotic plaques were deposited in aorta and aortic roots of mice. To examine whether the inflammatory responses occurred in mice aorta and aortic roots, the flow cytometry and immunofluorescence staining were performed. The CD45 was used as a marker of leukocytes and the aortic leukocytes (CD45^+^) may reflect the inflammatory activity. The results indicated that compared with arthritis mice (+ K/BxN serum −HFD), the aortic CD45^+^ leukocytes significantly increased in arthritis mice combined with atherosclerosis (+ K/BxN serum + HFD) (*P* < 0.01). Compared with normal mice (−K/BxN serum −HFD), the aortic CD45^+^ leukocytes significantly increased in atherosclerosis mice (−K/BxN serum + HFD) (*P* < 0.01) (Fig. 3A). Meanwhile, CD68 is known as a typical phagocytic marker and an indicator of macrophage activation, which give rise to atherogenesis by acting as a leukocyte chemoattractant. Our results showed that compared with arthritis mice (+ K/BxN serum −HFD), the aortic CD68^+^ macrophages significantly increased in arthritis mice combined with atherosclerosis (+ K/BxN serum + HFD) (*P* < 0.001) (Fig. 3B). But the difference between atherosclerosis mice and normal mice was no significance. It indicated that HFD generated greater inflammation of arthritis mice in aorta.

Aortic roots were collected and stained by immunofluorescent. The results showed that compared with arthritis mice (+ K/BxN serum −HFD), the mean IOD of CD11c^+^ cells significantly increased in arthritis mice combined with atherosclerosis (+ K/BxN serum + HFD) (*P* < 0.05). Compared with normal mice (−K/BxN serum −HFD), the mean IOD of CD11c^+^ cells significantly increased in atherosclerosis mice (−K/BxN serum + HFD) (*P* < 0.05) (Fig. 3C). Alike, the mean IOD of IgG similarly increased in arthritis mice combined with atherosclerosis (+ K/BxN serum + HFD) and atherosclerosis mice (−K/BxN serum + HFD) (*P* < 0.05). And compared with atherosclerosis mice (−K/BxN serum + HFD), the mean IOD of IgG similarly increased in arthritis mice combined with atherosclerosis (+ K/BxN serum + HFD) (*P* < 0.05) (Fig. 3D). We also found that the deposition areas of CD11c and IgG were almost overlapping with the oil red O positive area (Fig. 2A). The accumulations of CD11b^+^ dendritic cells and CD68^+^ macrophages had no significant differences. It has been reported that mesangial IgG deposition was associated with greater inflammation (Lai et al. 2022). This result suggested that IgG deposition in aortic root was associated with more sever inflammation. Many immune cells infiltrating in the aortic root may contribute to the plaques’ formation. These results suggested that HFD triggered greater inflammation in aortic roots of arthritis mice.

Systemic inflammation of mice was determined by the levels of inflammatory cytokines in serum. The results indicated that compared with arthritis mice (+ K/BxN serum −HFD), the levels of IL-6 (*P* < 0.05), Eotaxin (*P* < 0.01), G-CSF (*P* < 0.05), IL-12p40 (*P* < 0.05), MIP-1β (*P* < 0.0001), MIP-1α (*P* < 0.001) and MCP-1 (*P* < 0.05) significantly increased in arthritis mice combined with atherosclerosis (+ K/BxN serum + HFD). Compared with normal mice (−K/BxN serum −HFD), the levels of G-CSF (*P* < 0.01), IL-12p40 (*P* < 0.05), MIP-1β (*P* < 0.001), MIP-1α (*P* < 0.01) significantly increased in atherosclerosis mice (-K/BxN serum + HFD) (Fig. 3E). T helper 1 cell (Th1) cytokines (IFN-γ and IL-2), T helper 2 cell (Th2) cytokines (IL-4, IL-5, IL-9, IL-10, IL-13), IL-1α, IL-1β, IL-3, IL-12p70, GM-CSF, KC and RANTES had no significant difference (data not shown). It suggested that HFD aggravated the systemic inflammation of arthritis mice.

### HFD increased the abundance of gut *Burkholderiaceae* and *Rhodanobacter*, decreased the abundance of gut* Marvinbryantia* and *Lactobacillus*, altered the intestinal homeostasis in arthritis mice combined with atherosclerosis

**Fig. 4:**
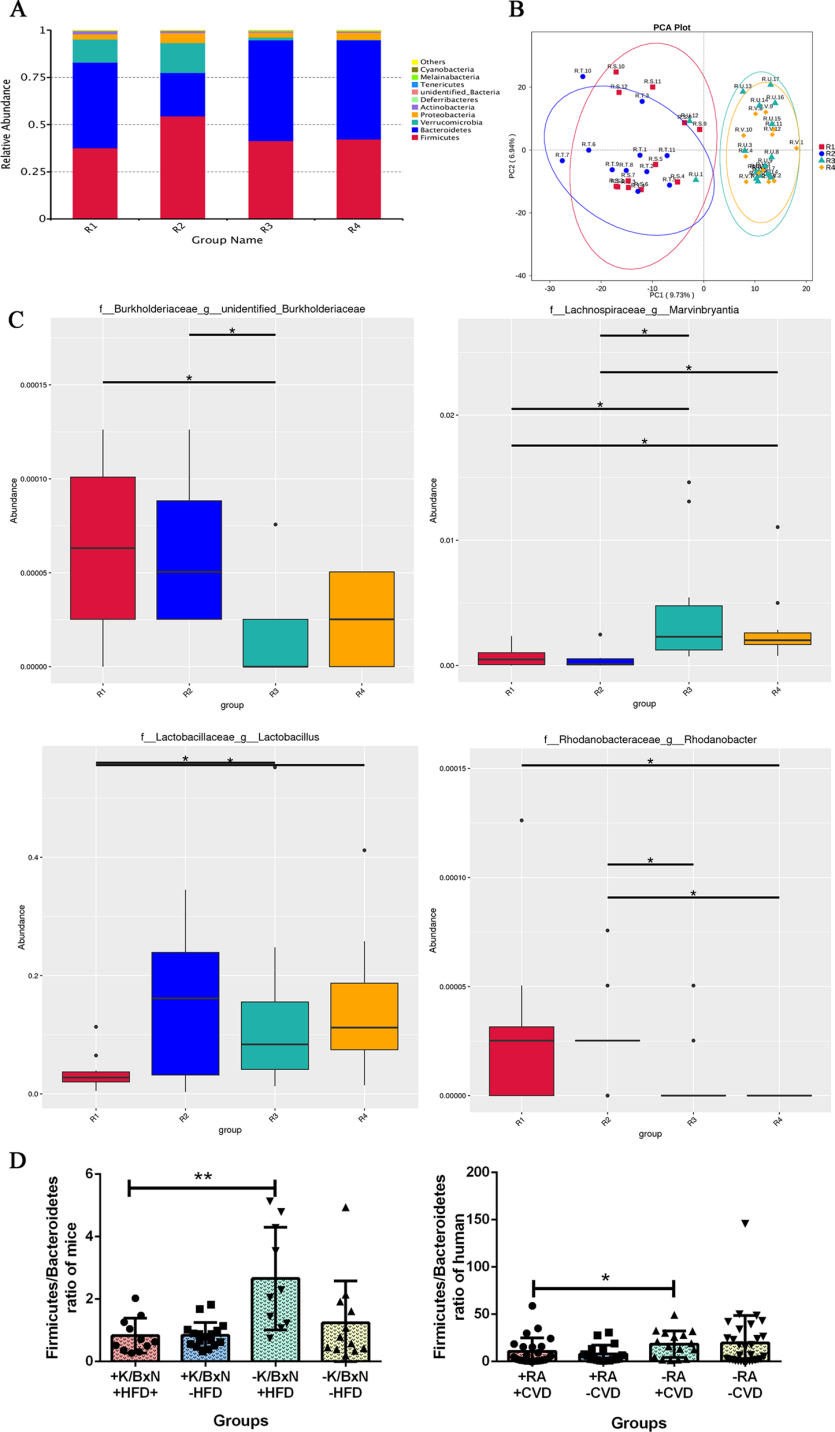
HFD increased the abundance of gut *Burkholderiaceae* and *Rhodanobacter,* decreased the abundance of gut *Marvinbryantia and Lactobacillus,* altered the intestinal homeostasis in arthritis mice combined with atherosclerosis. **A** Relative abundance of intestinal microbiota in four groups in phylum by 16S rDNA sequencing. **B** PCA plots of mice intestinal microbiota in four groups. **C** Comparison of different species among four groups in genus by MetaStat analysis. R1: arthritis mice combined with atherosclerosis (+ K/BxN serum + HFD), R2: atherosclerosis mice (−K/BxN serum + HFD), R3: arthritis mice (+ K/BxN serum-HFD), R4: normal mice (−K/BxN serum-HFD). **D** *Firmicutes/Bacteroidetes* ratio of mice and human gut microbiota in four groups. Data are presented as mean ± SD. Asterisk denotes statistically significant differences: *P < 0.05, **P < 0.01, ***P < 0.001. Representative results of two independent experiments are shown

To identify whether the gut microbiota of arthritis mice combined with atherosclerosis changed or not, we analyzed the gut microbial communities by 16S rDNA sequencing. The results showed that the gut microbiota constitution of arthritis mice combined with atherosclerosis (R1, + K/BxN serum + HFD), atherosclerosis mice (R2, −K/BxN serum + HFD) and arthritis mice (R3, + K/BxN serum-HFD) were mainly *Firmicutes*, *Bacteroidetes*, *Verrucomicrobia* and *Proteobacteria*, while the normal mice (R4, -K/BxN serum-HFD) mainly consist of *Firmicutes*, *Bacteroidetes* and *Proteobacteria* (Fig. 4A). The characteristic variables of fluorescence spectra are extracted by analyzing the major attributes which are showed by principal components analysis (PCA). The results indicated that the four groups were separated independently. Arthritis mice combined with atherosclerosis (R1, + K/BxN serum + HFD) and atherosclerosis mice (R2, −K/BxN serum + HFD) shared similar microbiota, whereas arthritis mice (R3, + K/BxN serum-HFD) and normal mice (R4, −K/BxN serum-HFD) had common microbiota (Fig. 4B). These results indicated that gut microbiota were heavily influenced by food. The MetaStat analysis by 16S rDNA sequencing displayed the species with significant difference between the two groups. It suggested that comparison of different species between two groups in genus, *Burkholderiaceae* in arthritis mice combined with atherosclerosis (R1, + K/BxN serum + HFD) were significantly higher than those of arthritis mice (R3, + K/BxN serum −HFD) (*P* < 0.05). *Marvinbryantia* and *Lactobacillus* in arthritis mice combined with atherosclerosis (R1, + K/BxN serum + HFD) were significantly lower than those of arthritis mice (R3, + K/BxN serum-HFD) ( *P* < 0.05) and normal mice (R4, −K/BxN serum-HFD) (*P* < 0.05) (Fig. 4C). The *Firmicutes*/*Bacteroidetes* ratio is significantly connected with the maintenance of intestinal homeostasis. The *Firmicutes*/*Bacteroidetes* ratio of arthritis mice combined with atherosclerosis (+ K/BxN serum + HFD) was significantly lower than that of atherosclerosis mice (−K/BxN serum + HFD) (Fig. 4D), the tendency is similar to the *Firmicutes*/*Bacteroidetes* ratio of human.

### The gut microbiota of RA patients combined with CVD were altered

**Fig. 5:**
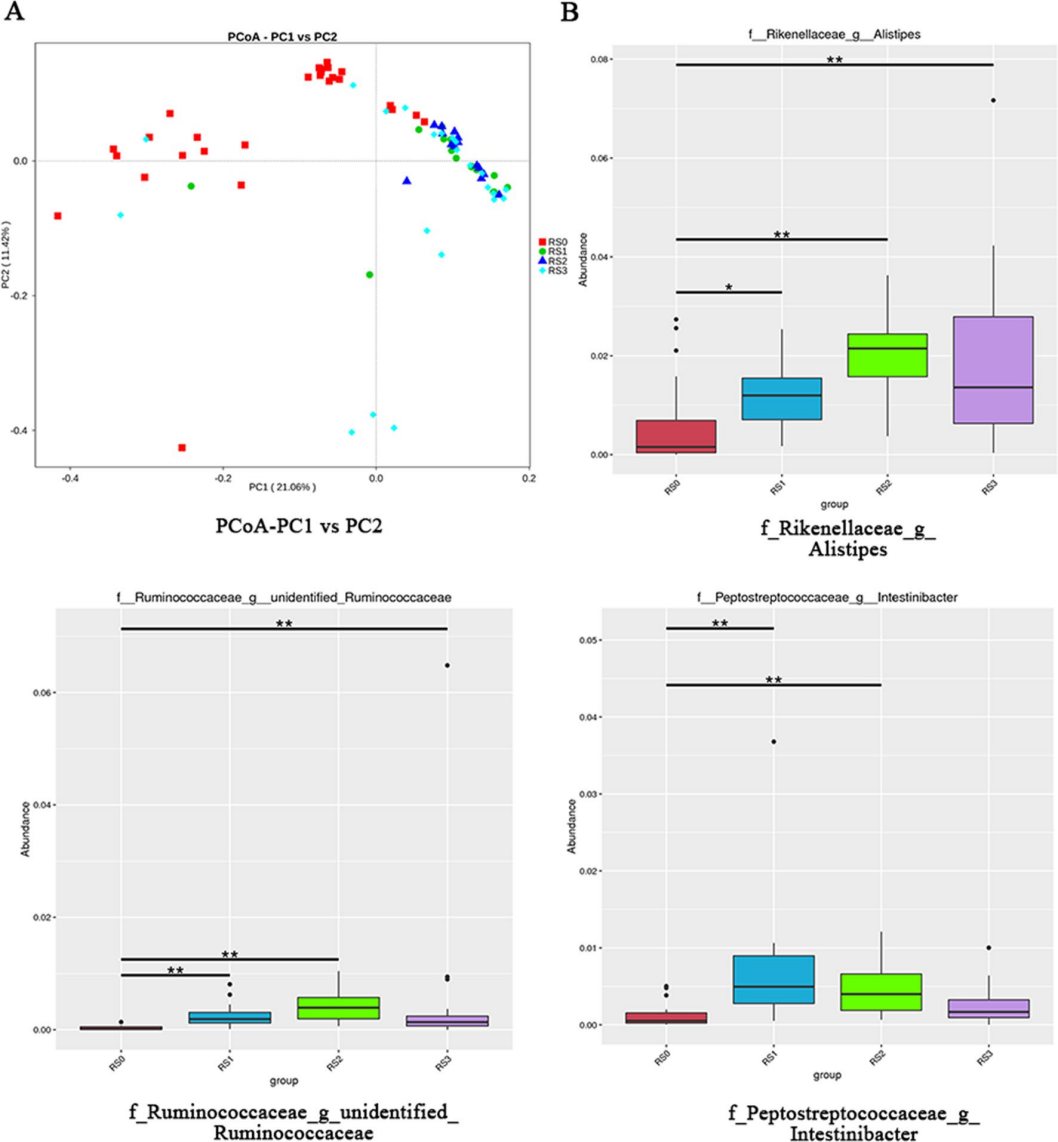
The gut microbiota of RA patients combined with atherosclerosis were altered. **A** Principal coordinate analysis (PCoA) among four groups of patients by 16S rDNA sequencing. **B** Multivariate statistical analysis of Metastat among four groups of patients. RS0: healthy human, RS1: RA patients, RS2: CVD patients, RS3: RA patients combined with CVD. Representative results of two independent experiments were shown

Faeces of RA patients, CVD patients, RA patients combined with CVD and healthy human were collected and analyzed by 16S rDNA sequencing. By principal coordinate analysis (PCoA), we visualized group differences, in which the microbiota of healthy human (RS0, healthy human) gathered together in red dots while the microbiota of other patients clustered in green (RS1, RA patients), blue (RS2, CVD patients) and light blue (RS3, RA patients combined with CVD) dots respectively (Fig. 5A). Multivariate statistical analysis of Metastat suggested that compared with healthy human (RS0), RA patients (RS1), CVD patients (RS2) and RA patients combined with CVD (RS3) all had significantly increased number of *Ruminococcaceae* and *Alistipes*, while only the RA patients combined with CVD (RS3) experienced no significant difference in *Intestinibacter* amount (Fig. 5B). It concluded that the gut microbiota of RA patients combined with CVD, RA patients and CVD patients were altered.

### HFD activated the gut metabolites of primary bile acid biosynthesis, aldosterone synthesis and secretion, and purine metabolism in arthritis mice combined with atherosclerosis

**Fig. 6:**
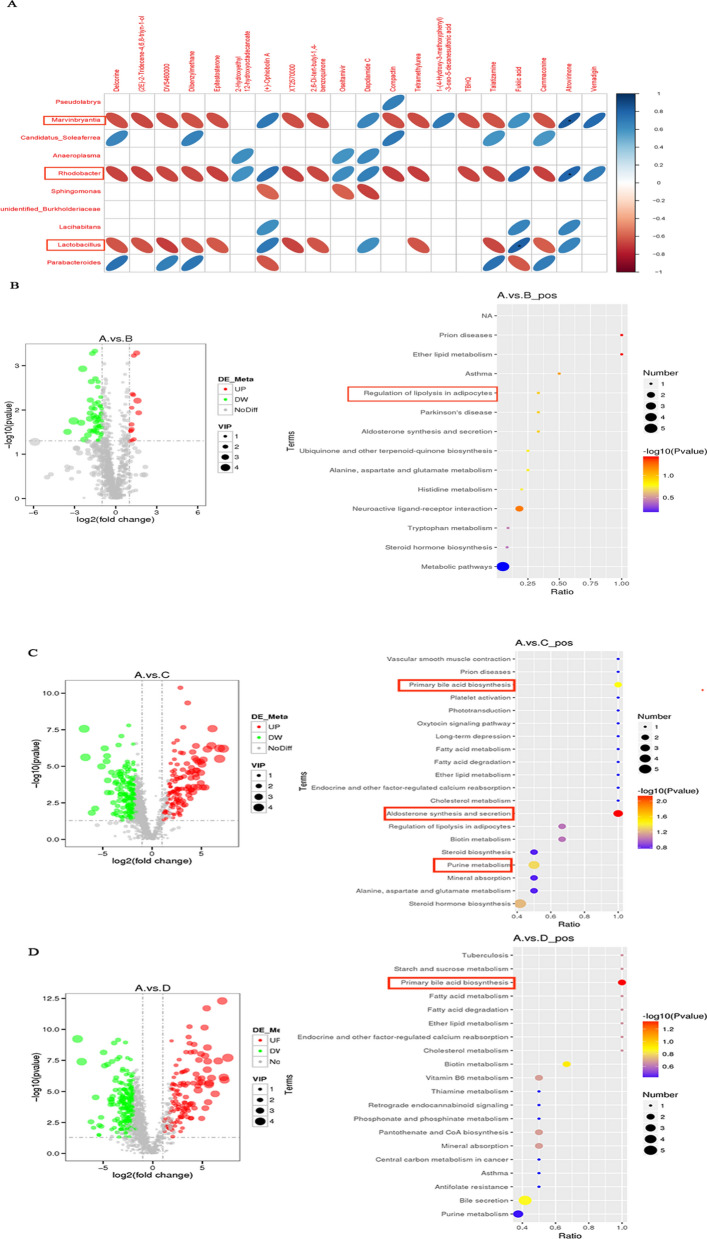
HFD activated the intestinal metabolites of primary bile acid biosynthesis, aldosterone synthesis and secretion, and purine metabolism in arthritis combined with atherosclerosis mice. **A** The association analysis between 16S sequencing and metabolites analysis. **B** Volcano plot visualizing metabolites separation and KEGG pathway enrichment analysis for common differential metabolites in the arthritis combined with atherosclerosis mice and atherosclerosis mice by metabolites analysis. **C** Volcano plot visualizing metabolites separation and KEGG pathway enrichment analysis for common differential metabolites in the arthritis combined with atherosclerosis mice and arthritis mice. **D** Volcano plot visualizing metabolites separation and KEGG pathway enrichment analysis for common differential metabolites in the arthritis combined with atherosclerosis mice and normal mice. In every figures “A” represented arthritis combined with atherosclerosis mice (+ K/BxN serum + HFD), “B” represented atherosclerosis mice (−K/BxN serum + HFD), “C” represented arthritis mice (+ K/BxN serum-HFD), “D” represented normal mice (−K/BxN serum-HFD). Red dots indicated the up-regulated metabolites, green dots indicated the down-regulated metabolites, gray dots indicated no significant difference in the figures of volcano plot. Representative results of two independent experiments were shown

By correlation analysis between 16S rDNA sequencing and metabolites analysis of mice faeces, we found that *Marvinbryantia*, *Lactobacillus* and *Rhodanobacter* were significantly correlated with several metabolites (Fig. 6A). Then we analyzed the related metabolic pathway of arthritis mice combined with atherosclerosis.

The results of metabolism analysis for mice faeces indicated that a total of 1242 differential metabolites were identified between the arthritis mice combined with atherosclerosis (A, + K/BxN serum + HFD) and atherosclerosis mice (B, −K/BxN serum + HFD) based on the standard of VIP > 1, FC > 2 or FC < 0.5 and *P* value < 0.05. 14 metabolites were up-regulated, while 50 metabolites were down-regulated. Then, we performed KEGG enrichment analysis in order to explore the potential function of these differential metabolites. The results suggested that these differential metabolites were mainly associated with the regulation of ether lipid metabolism (*P* value < 0.05) (Fig. 6B). We also analyzed 1242 differential metabolites between the arthritis mice combined with atherosclerosis (A, + K/BxN serum + HFD) and arthritis mice (C, + K/BxN serum-HFD) based on the same standards. The results indicated that there were 122 up-regulated metabolites, while 183 metabolites were down-regulated. KEGG enrichment analysis suggested that these differential metabolites mainly related to the regulation of aldosterone synthesis and secretion, primary bile acid biosynthesis and purine metabolism (*P* value < 0.05) (Fig. 6C). Between the arthritis mice combined with atherosclerosis (A, + K/BxN serum + HFD) and normal mice (D, -K/BxN serum-HFD), 192 of different metabolites were up-regulated, while 366 ones were down-regulated. KEGG enrichment analysis suggested that these differential metabolites mainly involved in the regulation of primary bile acid biosynthesis (*P* value < 0.05) (Fig. 6D). These results concluded that HFD activated the gut metabolites of primary bile acid biosynthesis, aldosterone synthesis and secretion, and purine metabolism in arthritis mice combined with atherosclerosis.

### HFD activated the colonic DEGs of IL-17 signaling pathway, increased the Th17 and decreased the Treg lamina propria lymphocytes in arthritis mice combined with atherosclerosis

**Fig. 7:**
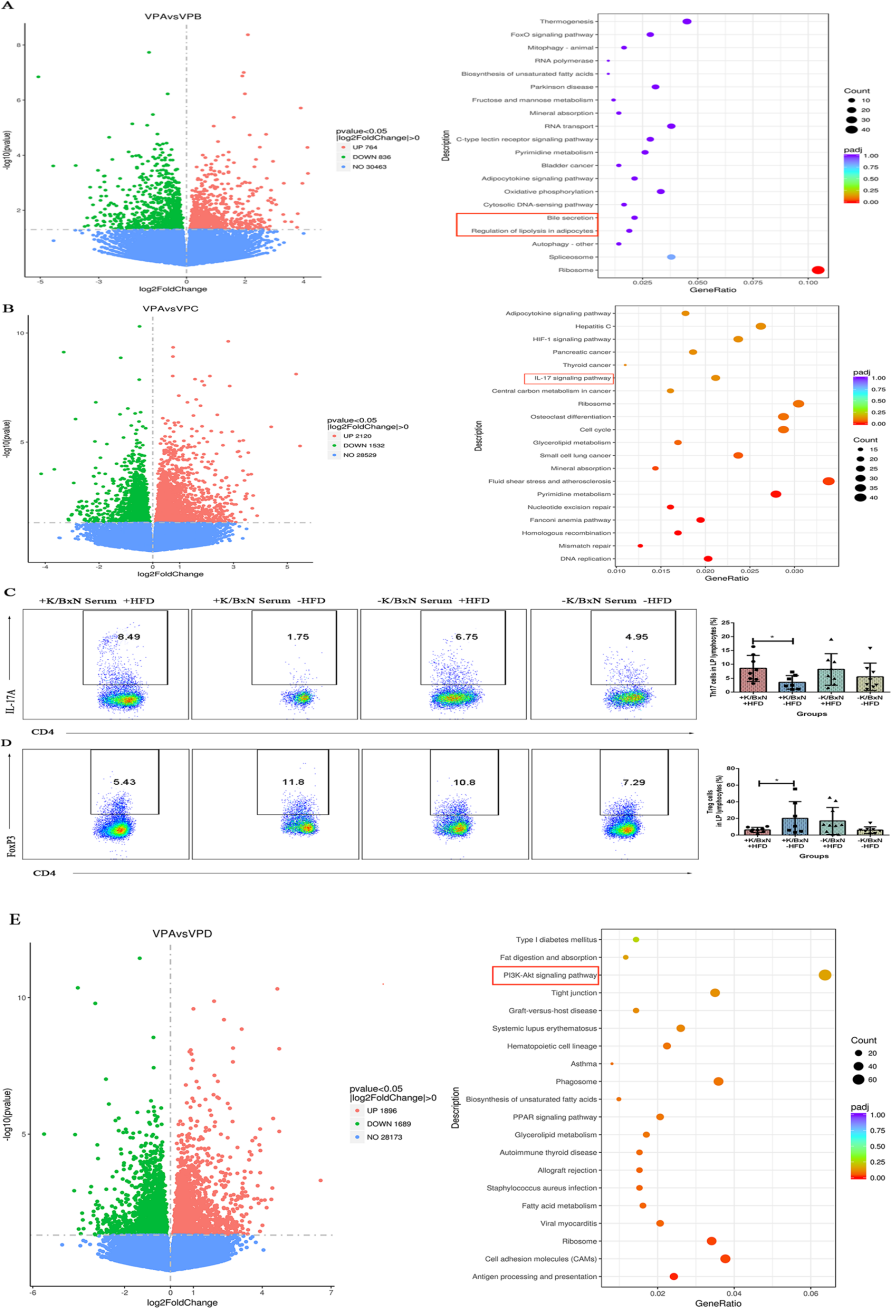
HFD activated the colonic DEGs of IL-17 signaling pathway, increased the Th17 and decreased the Treg lamina propria lymphocytes in arthritis combined with atherosclerosis mice. **A** Volcano plot visualizing differential expression genes in colons and KEGG pathway enrichment analysis in the arthritis combined with atherosclerosis mice and atherosclerosis mice. **B** Volcano plot visualizing differential expression genes in colons and KEGG pathway enrichment analysis in the arthritis combined with atherosclerosis mice and arthritis mice. **C** Flow cytometry gating strategy and the percent of Th17 cells (CD4^+^ IL-17A^+^). **D** Flow cytometry gating strategy and the percent of Treg cells (CD4^+^ FoxP3^+^). **E** Volcano plot visualizing differential expression genes in colons and KEGG pathway enrichment analysis in the arthritis combined with atherosclerosis mice and normal mice. VPA: arthritis combined with atherosclerosis mice (+ K/BxN serum + HFD), VPB: atherosclerosis mice (−K/BxN serum + HFD), VPC: arthritis mice (+ K/BxN serum-HFD), VPD: normal mice (−K/BxN serum-HFD). Representative results of two independent experiments were shown

In order to determine the differential expression genes (DEGs) of colon, we analyzed the mice colons by transcriptome sequencing. The results indicated that between arthritis mice combined with atherosclerosis (VPA, + K/BxN serum + HFD) and atherosclerosis mice (VPB, −K/BxN serum + HFD), there were 1600 DEGs (based on a threshold change of 1.3-fold or more, and adjusted for a q-value less than 0.05), with 764 up-regulated genes and 836 down-regulated ones. These DEGs mainly involved in bile secretion and regulation of lipolysis in adipocytes (P value < 0.05) (Fig. 7A). Among arthritis mice combined with atherosclerosis (VPA, + K/BxN serum + HFD) and arthritis mice (VPC, + K/BxN serum-HFD) were 3652 DEGs, with 2120 up-regulated genes and 1532 down-regulated genes, which mainly participated in IL-17 signaling pathway (*P* value < 0.05) (Fig. 7B). Flow cytometry analysis indicated that compared with arthritis mice (+ K/BxN serum-HFD), the amount of Th17 (CD4^+^IL-17^+^) lamina propria lymphocytes of arthritis mice combined with atherosclerosis (+ K/BxN serum + HFD) were significantly increased and Treg (CD4^+^FoxP3^+^) lamina propria lymphocytes were significantly decreased. However, compared with normal mice (−K/BxN serum-HFD), the amount of Th17 and Treg lamina propria lymphocytes of atherosclerosis mice (−K/BxN serum + HFD) showed no significant differences (Fig. 7C and D). These results concluded that HFD increased the amount of Th17 and decreased the Treg lamina propria lymphocytes of arthritis mice combined with atherosclerosis (+ K/BxN serum + HFD). In comparison with normal mice (VPD, −K/BxN serum −HFD), there were 3585 DEGs in arthritis mice combined with atherosclerosis, with 1896 up-regulated genes and 1689 down-regulated genes. These DEGs mainly clustered in PI3K-Akt signaling pathway (*P* value < 0.05) (Fig. 7E). It suggested that HFD activated the colonic DEGs of IL-17 signaling pathway, increased the Th17 and decreased the Treg lamina propria lymphocytes in arthritis mice combined with atherosclerosis, which are associated with inflammation.

### HFD decreased the number of Peyer’ s patches and the mature B cells in Peyer’ s patches, increased the B1 cells in arthritis combined with atherosclerosis mice

**Fig. 8:**
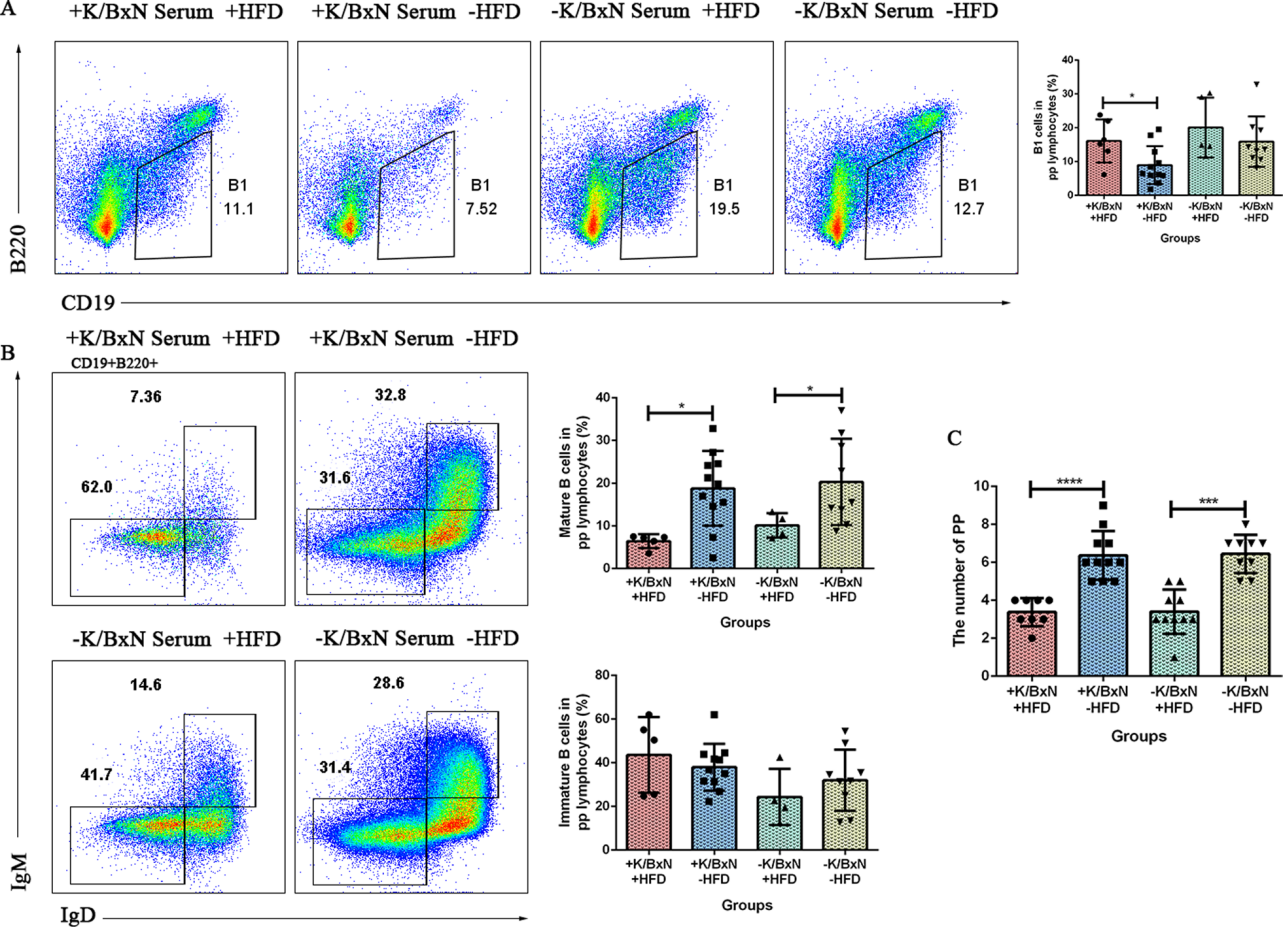
HFD decreased the number of Peyer’ s patches and the mature B cells in Peyer’ s patches, increased the B1 cells in arthritis combined with atherosclerosis mice. **A** Flow cytometry gating strategy and the percentage of B1 cells (CD19^+^B220^−^) of Peyer’s patches lymphocytes in four groups. **B** Flow cytometry gating strategy and the percentage of mature B cells (CD19^+^B220^+^IgD^+^IgM^+^) and immature B cells (CD19^+^B220^+^IgD^−^IgM^−^) of peyer’s patches lymphocytes in four groups. **C** The number of peyer’s patches in four groups. Data were represented as mean ± SD. Asterisk denoted statistically significant differences: *P < 0.05, **P < 0.01, ***P < 0.001. Representative results of two independent experiments were shown

The gut microbiota, metabolites and colonic DEGs of arthritis mice were all influenced by HFD. To identify whether the intestinal lymphocytes were influenced by HFD or not, Peyer’ s patches lymphocytes were analyzed by flow cytometry. The results indicated that compared with arthritis mice (+ K/BxN serum −HFD), arthritis mice combined with atherosclerosis (+ K/BxN serum + HFD) had significantly increased amount of B1 cells (CD19^+^B220^−^) (*P* < 0.05), decreased number of mature B cells (CD19^+^B220^+^IgD^+^IgM^+^) (*P* < 0.05) in Peyer’ s patches as well as the number of Peyer’ s patches (*P* < 0.0001). Compared with normal mice (−K/BxN serum −HFD), mature B cells (CD19^+^B220^+^IgD^+^IgM^+^) (*P* < 0.05) and the number of Peyer’ s patches (*P* < 0.001) in atherosclerosis mice (−K/BxN serum + HFD) significantly decreased, but the B1 cells and immature B cells (CD19^+^B220^+^IgD^−^IgM^−^) displayed no significant differences (Fig. 8A–C). It suggested that HFD disrupted the intestinal homeostasis of arthritis mice, which triggered the inflammatory responses.

### HFD increased the CD3^+^/CXCR3^+^ cells, CD68^+^ macrophages, CD3^+^ T cells in lamina propria lymphocytes in arthritis mice combined with atherosclerosis

**Fig. 9:**
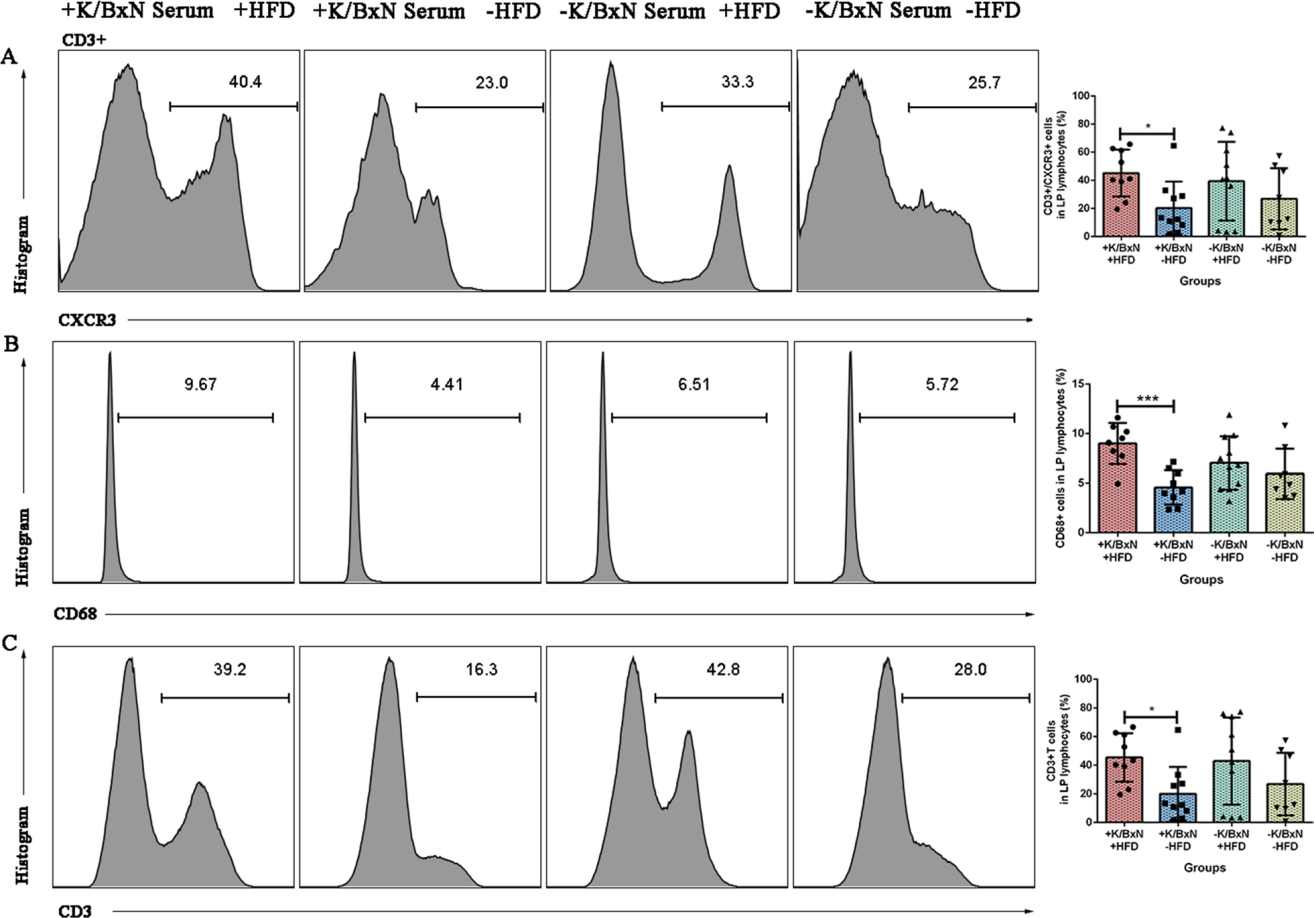
HFD increased the CD3^+^/CXCR3^+^ cells, CD68^+^ macrophages, CD3^+^ T cells in lamina propria lymphocytes in arthritis mice combined with atherosclerosis. **A** Flow cytometry gating strategy and the percentage of CD3^+^/CXCR3^+^ cells of lamina propria lymphocytes in four groups. **B** Flow cytometry gating strategy and the percentage of CD68^+^ macrophages of lamina propria lymphocytes in four groups. **C** Flow cytometry gating strategy and the percentage of CD3^+^ T cells of lamina propria lymphocytes in four groups. Data were represented as mean ± SD. Asterisk denoted statistically significant differences: *P < 0.05, **P < 0.01, ***P < 0.001. Representative results of two independent experiments were shown

To identify whether the intestinal lymphocytes were influenced by HFD or not, lamina propria lymphocytes were analyzed by flow cytometry. The results indicated that compared with arthritis mice (+ K/BxN serum −HFD), arthritis mice combined with atherosclerosis (+ K/BxN serum + HFD) significantly increased the CD3^+^/CXCR3^+^ cells (*P* < 0.05), CD68^+^ macrophages (*P* < 0.001), CD3^+^ T cells (*P* < 0.05). Compared with normal mice (−K/BxN serum -HFD), the populations of CD3^+^/CXCR3^+^ cells, CD68^+^ macrophages, CD3^+^ T cells of atherosclerosis mice (−K/BxN serum + HFD) did not exhibit any significant differences (Fig. 9A–C). All the results of lamina propria lymphocytes concluded that HFD increased the CD3^+^/CXCR3^+^ cells, CD68^+^ macrophages, CD3^+^ T cells in lamina propria lymphocyte in arthritis mice combined with atherosclerosis, altered the ratio of Th17/Treg, disrupted the intestinal homeostasis, and the intestinal immunity was influenced through the altered gut barrier function.

## Discussion

Both diseases of the arthritis and atherosclerosis are related with gut microbiota (Xu et al. 2022; Nesci et al. 2023; Jie et al. 2017; Lai et al. 2022; Qin et al. 2023). The gut microbiota may play a key role in the modulation of immune system and metabolic homeostasis. Some inflammatory diseases were induced by the dysbiosis of gut microbiota and the permeability of gut barrier, such as colitis-associated cancers and inflammatory bowel disease (Huang et al. 2022). Our results indicated that compared to healthy human (RS0), the gut microbiota of RA patients combined with CVD (RS3), RA patients (RS1) and CVD patients (RS2) were all altered. The genera of *g_Ruminococcaceae* and *g_Alistipes* which are both associated with CVD (Jiang et al. 2022; Ahrens et al. 2021; Dong et al. 2023; Neyrinck et al. 2019), increased significantly compared to healthy human. It is well known that *Alistipes* is a genus of bacteria under *Bacteroidetes*, which may associate with the modulation of inflammatory and secondary metabolites, and could produce lipopolysaccharide (LPS) which may cause systemic changes (Dhaliwal 2019). These results indicated that the microbiota changes may associate with the intricate interplay among the immune system, dietary factors, and the gut microbiota (Shao et al. 2023). The gut microbiota is known to be a dynamic community of microorganisms that plays a pivotal role in maintaining host health (Lai et al. 2021). However, disruptions in this microbial balance, termed dysbiosis, have been implicated in various diseases, including arthritis and atherosclerosis.

In order to confirm more kinds of alterations in the disease of RA combined with CVD, murine model of ApoE^−/−^ mice in combination with K/BxN serum and HFD was employed in our study. 16S rDNA sequencing of mice showed that arthritis mice combined with atherosclerosis had lower level of *g_Lactobacillus* and *g_Marvinbryantia*, and higher level of *g_Burkholderiaceae* and *g_Rhodanobacter*. Previous studies indicated that probiotic *Lactobacillus* could prevent the generation of atherosclerotic plaque through the inhibition of intestinal cholesterol absorption (Zhai et al. 2022; Huang et al. 2014). We found that the abundance of *g_Lactobacillus* significantly decreased in the intestinal tracts of arthritis mice combined with atherosclerosis while increased in atherosclerosis mice, which was consistent with previous studies and conformed that probiotic *Lactobacillus* could prevent the generation of atherosclerotic plaque (Dong et al. 2022). In addition, high salt intake induces the depletion of *g_Lactobacillus* and accession of blood pressure and CD4^+^ Rorγt^+^ Th17 cells (Dong et al. 2022). It was reported that increasing the abundance of *Lactobacillus* could induce the joint swelling and bone destruction (Lai et al. 2022). Our study indicated that the changes in microbial composition in the HFD group brought about severe atherosclerotic lesions and bone destruction, which indicated that the gut microbiota influenced the atherosclerosis and arthritis. In our study the abundance of the *g_Marvinbryantia* in arthritis mice combined with atherosclerosis was detected to be low, thus these mice exhibited intestinal inflammation and dysregulated homoeostasis. This result is consistent with previous study (Aarnoutse et al. 2022).

Specifically, the inflammatory milieu created by arthritis could alter the gut environment, making it more susceptible to dietary perturbations. Arthritis-related inflammation can disrupt the normal function of the gut, including alterations in gut permeability and intestinal homeostasis. These disruptions may create an environment conducive to microbial imbalances, particularly when challenged by dietary factors like HFD. The immune response of arthritis could also exert direct or indirect influences on the gut microbiota (Filippis et al. 2021). Cytokines and other immune mediators released during arthritis have been shown to affect the growth and survival of specific microbial species. These immune mediators may create a selective pressure that favors the proliferation of certain microbial taxa while inhibiting others, thereby shaping the gut microbiota profile (Zhou et al. 2021). This immune-mediated modulation of the gut microbiota could further exacerbate the inflammatory cascade, creating a vicious cycle that perpetuates disease progression.

Moreover, the changes in gut permeability and intestinal homeostasis demonstrated in our study could contribute to the observed microbiota differences. Arthritis-induced inflammation may disrupt the integrity of the intestinal barrier, leading to increased microbial translocation and altered microbial-host interactions. These disruptions could provide opportunities for microbiota dysbiosis, particularly under the influence of HFD. HFD is known to alter the gut microbiota, often leading to an increase in pathogenic species and a decrease in beneficial bacteria (Pan et al. 2023). In the context of arthritis, these HFD-induced changes may be more pronounced, leading to a more severe microbiota imbalance.

Interpreting these findings, it is plausible that the combined effects of arthritis and HFD create a unique gut environment that selects for specific microbial species or promotes the growth of opportunistic pathogens (Caruso et al. 2024; Araújo et al. 2024). The altered gut environment created by arthritis and HFD may provide an opportunity for these pathogens to proliferate, leading to microbiota dysbiosis. These changes could then feedback into the immune system, exacerbating the inflammatory cascade and promoting the development of atherosclerosis.

Our study showed that arthritis itself decreased the *Firmicutes/Bacteroidetes* ratio, sharing the similar tendency with that of human. Lower *Firmicutes/Bacteroidetes* ratio concluded that the microbiome homeostasis was disturbed, the dysbiosis was associated with local inflammatory response especially the Th17 responses (Luca and Shoenfeld 2018; Liu and Yanziyao 2024), and also related with weight loss, inflammatory bowel disease (IBD) and several pathological conditions (Magne et al. 2020; Mariat et al. 2009; Stojanov et al. 2020). IBD represents a complex inflammation in the small and large intestine (Stojanov et al. 2020). Our study indicated that the pathological condition of arthritis combined with atherosclerosis was in relation with the decreased level of *Firmicutes/Bacteroidetes* ratio, which involved in intestinal inflammation and imbalanced intestinal homeostasis.

The altered gut microbiota directly resulted to the change of metabolites, and it was found that HFD mainly mediated the metabolic pathway of primary bile acid biosynthesis. Primary bile acids, which consist of chenodeoxycholic acid (CDCA) and cholic acid (CA), are synthesized in the hepatocytes in humans, conjugated to taurine or glycine and then excreted into the intestine. Levels of primary bile acids were also elevated in the faeces of some RA patients (Su et al. 2023). The levels of primary bile acids were significantly increased, 3-oxo lithocholic acid (LCA) and isoLCA were signifificantly reduced in the gut of inflammatory bowel diseases (IBD) patients (Franzosa et al. 2019; Paik et al. 2022). The levels of 3-oxoLCA and isoLCA were inversely correlated with the expression of Th17 cell correlated genes (Paik et al. 2022). Bile acids and gut microbiota interact with each other. The synthesis and metabolism of bile acids are regulated by gut microbiota. Meanwhile, bile acids influence the composition of gut microbiota (Yang et al. 2021). Bile acids involved not only in metabolic disease, but also in CVD. They may contribute to atherosclerosis and endothelial dysfunction (Nesci et al. 2023). Bile acids metabolism is also promoted by the gut microbiota (Stojanov et al. 2020). Bile acids control host immune responses by modulating the Th17 and Treg balance directly and also modulate gut-associated events (Hang et al. 2019). In this regards, we supposed that the disordered gut microbiota impacted the bile acids which contribute to atherosclerosis，elevated the level of Th17 cells and decreased the Treg cells in lamina propria.

The lamina propria of the small intestine contains two large groups of CD4 T cells that regulate and balance the Th17/Treg cells (Cicerone and Pontone 2015). CD4^+^ T lymphocytes play a vital role in the pathogenesis of inflammatory diseases (Zhong et al. 2021). Th17 cells are marked by the cytokine of IL-17A and could stimulate the immune cells to produce pro-inflammatory cytokines, activate the cellular immune response that leads to the systemic inflammation (Qin et al. 2023). Treg cells generally inhibit the production of pro-inflammatory cytokines, and exhibit anti-inflammation activity (Qin et al. 2023) and are well-recognized for their indispensable role in maintaining immune homeostasis (Zaiss et al. 2021). They act as a natural brake on effector T cell responses, thereby preventing autoimmune diseases and fostering a balanced immune environment. Chronic inflammation associated with arthritis disrupts the delicate balance between Tregs and effector T cells, potentially leading to a breakdown in immune tolerance. This imbalance can exacerbate disease progression by allowing effector T cells to proliferate and cause tissue damage unchecked. Previous studies indicated that the composition and abundance of microbiota in the RA patients were strongly associated with the levels of CD4^+^ T cells subsets and Th17/Treg cytokines (Qin et al. 2023). One of the key pathogenesis of RA is the dysregulation of Th17/Treg cell in synovial, spleen, thymus tissues, peripheral blood, and the imbalance of CD4^+^ T lymphocyte subsets (Qin et al. 2023).

The significant changes in Treg populations between arthritis and non-arthritis mice were mostly attributed to HFD. Arthritis mice on an HFD exhibited more pronounced alterations in Treg populations compared to their non-arthritis counterparts. This suggests that the combination of arthritis and HFD created a synergistic effect on Treg homeostasis, likely due to the cumulative inflammatory burden. The HFD itself is known to induce metabolic stress and inflammation, which can further exacerbate the inflammatory milieu created by arthritis. This combined effect leads to a more significant dysregulation of Treg populations, potentially contributing to disease worsening. Decreased Treg amount in arthritis mice could impair their ability to control effector T cell responses, so that the sustained inflammation and tissue damage were promoted. This immune dysregulation may contribute to the aggravation of hyperlipidemia, atherosclerotic lesions, and arthropathy observed in our study. Tregs are known to regulate various immune cell populations, including macrophages, and their altered state could disrupt the entire immune network, leading to a cascade of pathological events (Tang et al. 2011).

Furthermore, Tregs have been shown to play a protective role in atherosclerosis by regulating macrophage activation and foam cell formation (Jiang et al. 2021). The altered Treg populations in arthritis mice, therefore, may exacerbate atherosclerosis progression by reducing their protective effects on the vascular wall. This finding underscores the complex interplay between Tregs, inflammation, and atherosclerosis, which suggested that restoring Treg function may be a therapeutic strategy to mitigate atherosclerosis in RA patients (Papadopoulou et al. 2012). In terms of disease progression, the dysregulation of Treg populations observed in our study represents a critical point of intervention (Song et al. 2020). Therapeutic strategies aimed at restoring Treg homeostasis could help mitigate the inflammatory burden and slow down the progression of both arthritis and atherosclerosis (Tong et al. 2021). Such strategies may include Treg-specific immunotherapy, dietary interventions that promote Treg function, or modulation of the gut microbiota, which has been shown to influence Treg populations. In conclusion, HFD altered the gut microbiota, intestinal homeostasis and Th17/Treg cell in lamina propria lymphocytes, and as a result the mucosal immunity was influenced through the altered gut barrier function (Dhaliwal 2019).

Mucosal immunity is based on an immunological homeostasis. When it is disrupted, the inflammation, allergy and infection are triggered by mucosal immunity (Takahiro kudo and Toshiaki shimizu 2023). Peyer' s patches contribute to the protect against pathogenic agents and lead to intestinal homeostasis (Garcıa-Pe~Na et al. 2017; Shou et al. 2021). The decreased number of Peyer's patches induced the pathogenic agents invasion and intestinal imbalance. It concluded that HFD disrupted the intestinal homeostasis of arthritis mice, which triggered the inflammatory responses. The flow cytometry of small intestinal cells indicated that HFD increased the percent of B1 cells, while decreased the percent of the mature B cells in Peyer's patches. It is known to all that the lamina propria lymphocytes are important for understanding the cellular and immune responses in the gut (Weigmann et al. 2007b).

Furthermore, our study suggested that the alterations in gut microbiota and metabolism induced by HFD could indirectly affect B cell development and function. Gut microbiota dysbiosis, which is often observed in RA patients and animal models, has been shown to influence systemic immunity, including B cell responses. The gut microbiota plays a crucial role in maintaining immune homeostasis, and disruptions in this balance can lead to immune dysregulation. Therefore, the observed changes in B cell populations might be a consequence of the disrupted gut-immune axis in our arthritis mice combined with atherosclerosis.

The DEGs of colons indicated that HFD activated the colonic DEGs of IL-17 signaling pathway. IL-23/IL-17 axis contributes to the development of RA (Li and Tsokos 2021). IL-17 is a multifunctional pathway and it involves in up-regulating inflammatory gene expressions, activating NF-κB and induces pro-inflammatory cytokines (such as IL-6, TNF-α and IL-1), T cell and neutrophil-attracting chemokines including CCL-2, CCL-7, CXCL-1 and CXCL-2, destructing bones which observed in the models of arthritis, regulating several genes which restricted to gut epithelia and contributing to maintenance of intestinal barrier integrity (Li and Tsokos 2021; Amatya et al. 2017).

Phosphoinositide 3-kinase/protein kinase B (PI3K-AKT) signaling pathway is an intracellular signaling pathway which is associated with glucose metabolism, enzymatic biological effects (Xie et al. 2019), cell proliferation, lipid metabolism and protein synthesis. The synovial fibroblasts in RA patients may be regulated by high expression of PI3K in the synovial tissue, which leads to TNF-α-mediated cartilage destruction and inflammatory erosive arthritis (Liu et al. 2021). This was confirmed in our study that PI3K-Akt signaling pathway was enhanced in arthritis mice combined with atherosclerosis mice and the cartilage destruction of these mice was aggravated. Thus, PI3K-AKT signaling pathway may be associated with aortic and systemic inflammation of mice.

The inflammatory response is initiated by immune system when certain gut bacterial species adhered to the gut mucosa and invade mucosal epithelial cells. Such inflammatory responses include the activation of monocytes and macrophages. TNF-α, released by these immune cells, regulates a variety of physiological processes, such as inflammatory response, activation and proliferation of immune cells. In our study, aortic CD45^+^ leukocytes and CD68^+^ macrophages significantly increased in arthritis mice combined with atherosclerosis and atherosclerosis mice. Macrophages promote atherosclerosis through recruitment of leukocytes and vascular smooth muscle cells, and contribute to the plaque instability (Gils et al. 2012). Thus, HFD increased aortic leukocytes and macrophages, then aggravated the atherosclerosis in aorta. HFD also increased the deposition of CD11c and IgG in aortic root. IgG deposition at the same location of atherosclerotic plagues was also increased by arthritis and which was associated with severer inflammation. What’s more, the deposition of IgG and monocytes/macrophages are required for the development of arthritis (Qiao et al. 2020) and atherosclerotic plagues. Many immune cells infiltrating in the aortic root may contribute to the plaque formation. These results suggested that HFD brought out greater inflammation in mice aorta and aortic root and are in accordance with previous research (Huang et al. 2022).

HFD exacerbated the aortic inflammation of mice. To identify its influence on systemic inflammation, we compared the levels of serum cytokines in four groups by luminex. HFD significantly increased the levels of IL-6, Eotaxin, IL-12p40, G-CSF, MCP-1, MIP-1α and MIP-1β. These cytokines and chemokines were all concerned with inflammation. IL-12p40 is essential for pathologies of bone disorders, rheumatoid arthritis and osteoarthritis. IL-12p40 deletion in mice protected bone mass along with aging-induced inflammation (Xu et al. 2020). G-CSF is a growth factor with pro-inflammatory effect (He et al. 2021), which could induce carotid inflammation (Singh et al. 2022), mediate inflammation-associated erythropoiesis dysfunction in bone marrow (Jing et al. 2020), and associated with chronic inflammation (He et al. 2021). MIP-1α and MIP-1β are the related members of the C–C chemokine subfamily. They are both crucial for immune responses (Ren et al. 2010), and exhibit pro-inflammatory activity (Cook 1996; Driscoll 1994). Thus, we suggested that HFD worsened both the local and systemic inflammation of mice.

Between arthritis mice combined with atherosclerosis (+ K/BxN serum + HFD) and arthritis mice (+ K/BxN serum-HFD), there were significant differences, however, between atherosclerosis mice (−K/BxN serum + HFD) and normal mice (−K/BxN serum-HFD), the differences were not significant, including aortic CD68^+^ macrophages, serum cytokines of Eotaxin, MCP-1 and IL-6, B1 cells of PP, Th17 cells, Treg cells, CD3^+^/CXCR3^+^ cells, CD68^+^ macrophages, CD3^+^ T cells of LP. Although the variate were both the HFD in two cases, but the presence of arthritis make the differences more significant. Arthritis mice combined with atherosclerosis (+ K/BxN serum + HFD) had most CD68^+^ macrophages infiltrated in aorta and intestinal, promoted the sustained inflammatory responses in aorta and intestinal (Lou et al. 2024). The activation of immune cells, particularly macrophages and T-cells, within the vessel wall (Libby 2021) may trigger the production of inflammatory cytokines and reactive oxygen species, which promote the formation and progression of atherosclerotic plaques (Liang et al. 2022). Furthermore, the chronic inflammatory state in arthritis may lead to the activation of the coagulation cascade, increasing the risk of atherosclerosis.

Eotaxin, MCP-1 and IL-6 were all associated with inflammation. Studies had shown that elevated levels of inflammatory cytokines, such as IL-6 and TNF-α, are predictive of future cardiovascular events in RA patients (Lou et al. 2024; Lavillegrand et al. 2024). Moreover, these cytokines have been implicated in the disruption of endothelial function, promotion of platelet aggregation, and acceleration of atherosclerosis. IL-6, as a pro-inflammatory cytokine, was considered to be a marker of inflammatory diseases. It has been shown that IL-6 plays a key role in autoimmune diseases (Yao et al. 2014). Eotaxin is central to allergic inflammation (Conroy et al. 1997). MCP-1 mediated inflammation (Xu et al. 2019), by up-regulating in smooth muscle cells in atherosclerosis and synoviocytes of rheumatoid arthritis (Palomino and Marti 2015).

Increased Th17/Treg, CD3^+^/CXCR3^+^ cells and CD3^+^ T cells of LP suggested that HFD influenced the gut immune responses and inflammatory responses. They all concluded that combined the + K/BxN serum and + HFD triggered more inflammatory responses. The arthritis induced inflammation which was mediated by a complex interplay of cytokines, chemokines, and immune cells (Kong et al. 2022; Wolf and Ley 2019). This inflammatory milieu not only exacerbates joint pathology but also promotes endothelial dysfunction, oxidative stress, and plaque formation in the arteries, thereby accelerating the atherosclerotic process (Bäck et al. 2019). Furthermore, our findings, which demonstrate that HFD aggravates arthritis and atherosclerosis, may be partially explained by the pro-inflammatory state inherent to arthritis. The HFD, by altering the gut microbiota and intestinal permeability, could exacerbate this inflammatory state, creating a vicious cycle where inflammation fuels both joint disease and cardiovascular pathology. The gut microbiota, often referred to as the "forgotten organ," plays a crucial role in modulating systemic inflammation, and its dysregulation in response to HFD could exacerbate the already heightened inflammatory state in arthritis mice.

## Conclusion

The ApoE^−/−^ mice which treated by K/BxN serum and HFD were successfully simulated arthritis combined with atherosclerosis and developed severe, destructive arthropathy. HFD aggravated the ankle swelling and the damage of articular cavity, and increased the symptoms of atherosclerosis. This study suggested that HFD altered the gut microbiota and metabolism, and the disordered gut microbiota may trigger the dysregulation of intestinal immune homeostasis. The pro-inflammatory molecules were allowed into the bloodstream when the intestinal permeability was altered on account of the microbiota dysbiosis which led to the up-regulation of the local and systemic inflammation. Our study highlighted the importance of considering the gut-immune axis in the pathogenesis of arthritis and atherosclerosis. Future research should aim to further elucidate the mechanisms underlying these observed microbiota changes and explore potential therapeutic interventions that target the gut microbiota to mitigate the adverse effects of arthritis and atherosclerosis risk.

## Data Availability

No datasets were generated or analysed during the current study.
